# Genetic changes involving the coral gastrovascular system support the transition between colonies and bailed-out polyps: evidence from a *Pocillopora acuta* transcriptome

**DOI:** 10.1186/s12864-021-08026-x

**Published:** 2021-09-26

**Authors:** Po-Shun Chuang, Satoshi Mitarai

**Affiliations:** Okinawa Institute of Science and Technology, 1919-1 Tancha, Onna-son, Kunigami-gun , 904-0495 Okinawa, Japan

**Keywords:** Coloniality, Polyp bail-out, ACE, ECE, Stony coral, Transcriptome

## Abstract

**Background:**

A coral colony is composed of physiologically integrated polyps. In stony corals, coloniality adopts a wide diversity of forms and involves complex ontogenetic dynamics. However, molecular mechanisms underlying coloniality have been little studied. To understand the genetic basis of coloniality and its contribution to coral ecology, we induced polyp bail-out in a colonial coral, *Pocillopora acuta*, and compared transcription profiles of bailed-out polyps and polyps in normal colonies, and their responses to heat shock and hyposalinity.

**Results:**

Consistent with morphological formation of a gastrovascular system and its neural transmission and molecular transport functions, we found genetic activation of neurogenesis and development of tube-like structures in normal colonies that is absent in bailed-out polyps. Moreover, relative to bailed-out polyps, colonies showed significant overexpression of genes for angiotensin-converting enzymes and endothelin-converting enzymes. In response to hyperthermal and hyposaline treatments, a high proportion of genetic regulation proved specific to either bailed-out polyps or colonies. Elevated temperatures even activated NF-κB signaling in colonies. On the other hand, colonies showed no discernible advantage over bailed-out polyps in regard to hyposalinity.

**Conclusions:**

The present study provides a first look at the genetic basis of coloniality and documents different responses to environmental stimuli in *P. acuta* colonies versus those in bailed-out polyps. Overexpression of angiotensin-converting enzymes and endothelin-converting enzymes in colonies suggests possible involvement of these genes in development of the gastrovascular system in *P. acuta*. Functional characterization of these coral genes and further investigation of other forms of the transition to coloniality in stony corals should be fruitful areas for future research.

**Supplementary Information:**

The online version contains supplementary material available at 10.1186/s12864-021-08026-x.

## Background

Colonial corals comprise physiologically integrated polyps generated through budding or fusion of discrete colonies [1–4]. During evolution of scleractinian corals (Class Anthozoa, Phylum Cnidaria), colonial lifestyles have appeared various times, suggesting evolutionary advantages to coloniality, at least in some environments [5]. In comparison to a solitary lifestyle, colony formation requires neurological communication and efficient transport of resources between integrated polyps [6–8]. This physiological integration is thought to facilitate coral recovery from local damage, as well as responses to environmental stimuli [9–11]. In addition, adoption of asexual reproduction for colonial growth enables constant addition and replacement of polyps in a colony, allowing some colonial corals, such as the deep-sea corals *Gerardia sp.* and *Leiopathes sp.*, to achieve virtually indefinite growth [12].

In stony corals, coloniality adopts a wide diversity of forms, ranging from low integration with little or no live connecting tissue to a morphological continuum with seemly indistinguishable polyp boundaries [13]. Typical ontogeny of colonial corals follows a solitary-to-colonial morphological transition, achieved through repeated budding. Solitary polyps are then generated from adult colonies via sexual reproduction, such as broadcast spawning or brooding, forming a complete life cycle [14]. However, coloniality sometimes reverts in different forms during the life cycle. Fragmentation of adult colonies generally results in smaller colonies, but can sometimes yield solitary polyps, especially in corals of a phaceloid growth form, such as the octopus coral, *Galaxea fascicularis*. Some corals, such as the lobed cactus coral, *Lobophyllia corymbosa*, develop an interconnected colony during early post-metamorphosis and transform into closely packed, but morphologically isolated polyps at a later stage [15]. In addition, under certain environmental conditions, some colonial corals can also revert to solitary lifestyles via retraction or degradation of coenosarc tissue [16–18]. Many previous studies have attempted to compare genetics of coral larvae or primary polyps to those of adult colonies [19–21]. However, as earlier studies focused mostly on metamorphosis or initiation of calcification, the molecular foundation underlying coral coloniality is still poorly understood.

Polyp bail-out is a coral stress response featuring loss of coloniality and detachment of individual polyps [22, 23]. Since its first recognition in late twentieth century [22], induction of polyp bail-out has been demonstrated using a variety of stimuli, such as calcium deprivation [24–26], hyperosmosis [23, 27, 28], elevated temperatures [29], and insecticides [30]. More recent studies have also shown that under suitable conditions, bailed-out polyps can be cultivated in a solitary form in the laboratory and that they can regenerate polyp morphology typical of colonial polyps, genetically recovering basic biological processes, such as metabolism and immune activity [25, 26, 28]. This coral stress response thus provides an opportunity to study the biology of coloniality from a perspective other than the usual transition to coloniality in stony corals.

To better understand colony formation in stony corals from a genetic perspective, in this study, we induced polyp bail-out in *Pocillopora acuta*, a reef-building coral commonly inhabiting tropical waters of the Indo-Pacific Ocean. Transcriptional profiles of bailed-out polyps and of normal colonies were examined under ambient and stressful environmental conditions (elevated temperature, intensified illumination, and hyposalinity). The results reveal different molecular fingerprints and stress responses in bailed-out polyps and normal colonies of *P. acuta*, providing the first genetic dataset for a discussion of the molecular biology of coral coloniality.

## Results

### Bailed-out polyps vs. normal colonies under ambient conditions

**Fig. 1:**
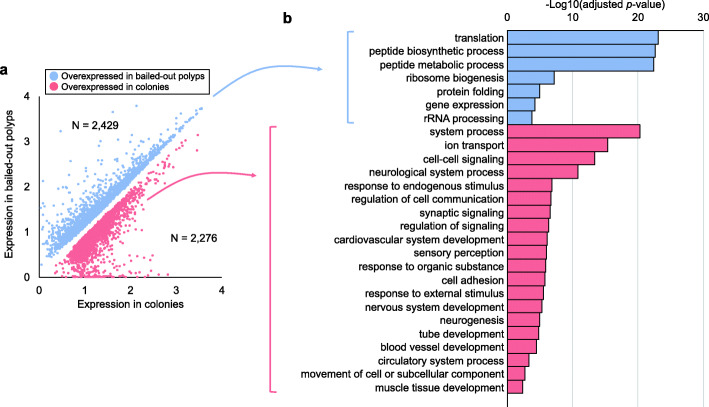
Genetic differentiation between bailed-out polyps and colonies under ambient conditions. (**a**) DEGs between bailed-out polyps and colonies. Expression levels are presented as transcripts per million (TPM) and are log-transformed with one unit shift to the right (log(TPM + 1)). DEGs significantly overexpressed in bailed-out polyps (blue; N = 2,429) and in colonies (red; *N* = 2,276) are labeled. (**b**) Selected GO terms overrepresented among DEGs overexpressed in bailed-out polyps (blue) and colonies (red)

Using Illumina RNAseq datasets from *P. acuta* under various cultivation conditions (i.e., ambient conditions, hypersalinity, hyperthermal, hyper-illumination, and hyposalinity) and of different morphotypes (i.e., colonies and bailed-out polyps), we first constructed a meta-transcriptome assembly of *P. acuta* (GenBank accession: GJAW00000000). Transcriptional profiles of normal colonies and of bailed-out polyps under ambient conditions were subjected to a differential gene expression (DE) analysis, which identified 4,705 differentially expressed genes (DEG) between bailed-out polyps and colonies (Fig. 1a; A complete list of DEGs is provided in Additional file 1). Among DEGs overexpressed in bailed-out polyps (*N* = 2,429), significant Gene Ontology (GO) enrichment occurred mostly in processes related to protein homeostasis, such as *translation* and *protein folding*. For DEGs overexpressed in colonies (*N* = 2,276), a more diverse array of GO categories was overrepresented, including *system process*, *ion transport*, *cell-cell signaling*, *cell adhesion*, *neurogenesis*, *tube development*, *movement of cell or subcellular component*, and *responses to endogenous and external stimuli* (Fig. 1b; A complete list of enriched GO categories is provided in Additional file 2).

**Fig. 2:**
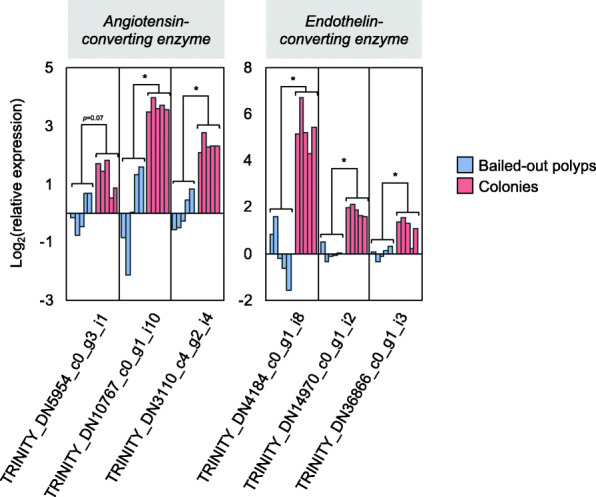
Expression of ACEs and ECEs in colonies (red) and bailed-out polyps (blue) based on a qPCR assay. Expression is presented as ΔCT (CT_average of control genes_ – CT_target gene_), normalized to the average ΔCT of bailed-out polyps. Statistically significant differences between colonies and bailed-out polyps (*p*-value < 0.05) are indicated by *

Among DEGs overexpressed in colonies, enrichment of *endocrine process (GO:0050886)* was marginally significant (Bonferroni-adjusted *p*-value = 0.045; Additional file 2). In this GO category, we found three transcripts functionally annotated as angiotensin-converting enzymes (ACE) and three as endothelin-converting enzymes (ECE). A quantitative polymerase chain reaction (qPCR) assay further confirmed overexpression of these genes in colonies relative to expression levels in bailed-out polyps, except for one ACE-like gene, which showed a marginally significant difference (*p* = 0.07) between bailed-out polyps and colonies (Fig. 2).

**Fig. 3:**
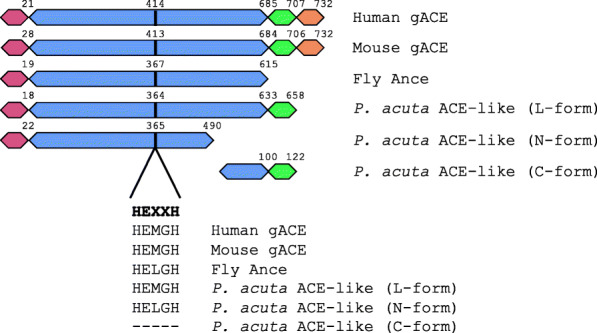
Domain structure of *P. acuta* ACE-like proteins. ACE homologs in human (AAA60611), mouse (AAA37149), and fly (AAB02171) are displayed for comparison. Signal peptide, cytoplasmic, transmembrane, and extracellular domains are indicated in red, orange, green, and blue, respectively. Sequences in a conserved *HEXXH* motif are presented. Ending position of each domain and starting position of the motif are indicated. The three *P. acuta* ACE-like proteins are indicated as L-form (TRINITY_DN5954_c0_g3_i1), N-form (TRINITY_DN10767_c0_g1_i10), and C-form (TRINITY_DN3110_c4_g2_i4)

Using the TransDecoder package in Trinotate, complete open reading frames (ORF) were predicted in all three ACE-like genes (ORF sequences are provided in Additional file 3). Sequence alignment and domain analysis showed that one *P. acuta* ACE-like protein (TRINITY_DN5954_c0_g3_i1) presented a structure similar to ACEs in other organisms, except for the absence of an intracellular domain (herein referred as L-form *P. acuta* ACE-like protein). The other two ACE-like proteins were shorter and aligned with the N-terminus (TRINITY_DN10767_c0_g1_i10; N-form) or C-terminus (TRINITY_DN3110_c4_g2_i4; C-form) of known ACEs, respectively (Fig. 3). A *HEXXH* motif was found in the L- and N-forms *P. acuta* ACE-like proteins, but not in the C-form. When compared with ACE proteins in other organisms, the three *P. acuta* ACE-like proteins showed 25–41% sequence identities (Table 1).

**Table 1:** Sequence identities between *P. acuta* ACE-like proteins and ACE homologs in other organisms. The three *P. acuta* ACE-like proteins are L-form (TRINITY_DN5954_c0_g3_i1), N-form (TRINITY_DN10767_c0_g1_i10), and C-form (TRINITY_DN3110_c4_g2_i4). Reference sequences were downloaded from the NCBI protein database (human: AAA60611; mouse: AAA37149; fly: AAB02171)

	Human	Mouse	Fly	N-form	C-form
Mouse	79.0%				
Fly	35.5%	35.1%			
N-form	41.2%	40.0%	40.7%		
C-form	25.3%	28.5%	32.8%	N/A	
L-form	37.1%	36.1%	36.4%	52.5%	41.6%

**Fig. 4:**
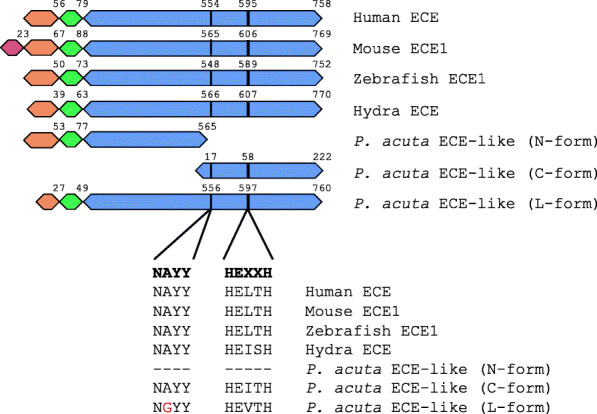
Domain structure of *P. acuta* ECE-like proteins. ECE homologs in human (BAA07800), mouse (NP_001356106), zebrafish (NP_001071260), and hydra (AAD46624) are displayed for comparison. Signal peptide, cytoplasmic, transmembrane, and extracellular domains are indicated in red, orange, green, and blue, respectively. Sequences in conserved *NAYY* and *HEXXH* motifs are presented. An Ala-to-Gly substitution was found in one *P. acuta* ECE-like protein. Ending position of each domain and starting position of each motif are indicated. The three *P. acuta* ECE-like proteins are indicated as L-form (TRINITY_DN14970_c0_g1_i2), N-form (TRINITY_DN4184_c0_g1_i8), and C-form (TRINITY_DN36866_c0_g1_i3)

For the three ECE-like genes, two were predicted with complete ORFs while one (TRINITY_DN3110_c4_g2_i4) showing an ORF missing a starting codon, likely due to incomplete assembly (ORF sequences are provided in Additional file 4). Alignment and domain analysis revealed that a *P. acuta* ECE-like protein (TRINITY_DN14970_c0_g1_i2) was structurally similar to full-length ECEs in other organisms (denominated the L-form *P. acuta* ECE-like protein), while the other two ECE-like proteins aligned with the N-terminal (TRINITY_DN4184_c0_g1_i8; N-form) and C-terminal parts (TRINITY_DN36866_c0_g1_i3; C-form) of known ECE proteins, respectively (Fig. 4). A *HEXXH* motif was found in the L- and C-forms of *P. acuta* ECE-like proteins. A *NAYY* motif was found only in the C-form, while in the L-form *P. acuta* ECE-like protein, the motif presented a substitution of Ala to Gly. When compared with ECE proteins in other organisms, the *P. acuta* ECE-like proteins showed sequence identities ranging from 23 to 60% (Table 2).

**Table 2:** Sequence identities between *P. acuta* ECE-like proteins and ECE homologs in other organisms. The three *P. acuta* ECE-like proteins are L-form (TRINITY_DN14970_c0_g1_i2), N-form (TRINITY_DN4184_c0_g1_i8), and C-form (TRINITY_DN36866_c0_g1_i3). Reference sequences were downloaded from the NCBI protein database (human: BAA07800; mouse: NP_001356106; zebrafish: NP_001071260; hydra: AAD46624)

	Human	Mouse	Zebrafish	Hydra	N-form	C-form
Mouse	91.0%					
Zebrafish	65.1%	65.5%				
Hydra	34.5%	34.3%	33.4%			
N-form	23.7%	25.1%	23.1%	23.1%		
C-form	59.9%	59.0%	55.9%	58.6%	N/A	
L-form	32.3%	32.0%	31.9%	31.7%	20.8%	56.3%

### Stress responses in bailed-out polyps and colonies

**Fig. 5:**
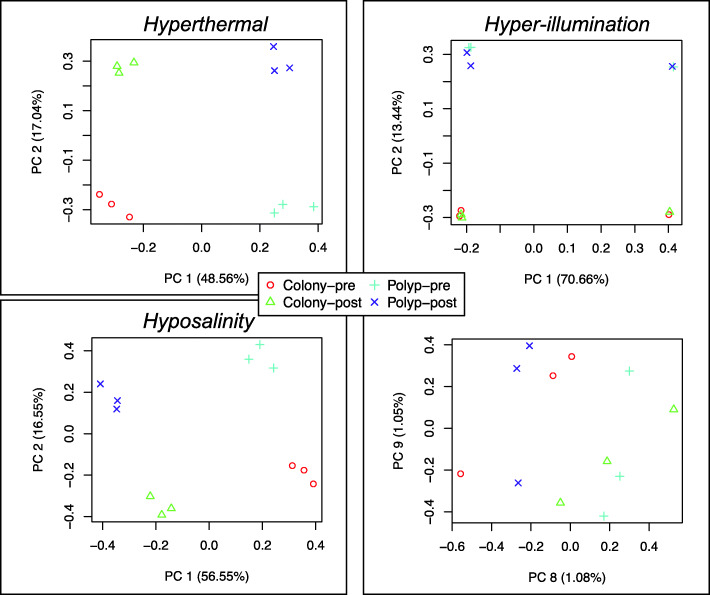
Principle component analyses (PCA) of bailed-out polyps and colonies in hyperthermal, hyposaline, and hyper-illuminated experiments. All three PCAs were based on the whole *P. acuta* transcriptome assembly (GenBank accession: GJAW00000000; N = 65,935 transcripts). Clear differences between pre- and post-treatment samples are reflected by the first two principle components (PC) in each analysis, except that for the hyper-illuminated experiment, in which the differences between pre- and post-treatment samples contribute only around 2% variations in the whole transcriptome assembly (PC8 and PC9)

To investigate whether bailed-out polyps and normal colonies differ in responses to external stimuli, we further examined genetic changes that occurred in response to hyperthermal (30 °C), hyposaline (25‰), and hyper-illumination (500 µmol/m^2^/s) treatments. Both hyperthermal and hyposaline conditions induced significant genetic responses in both bailed-out polyps and colonies (Fig. 5). Under intense lighting, genetic changes were relatively small in both groups (Fig. 5). Therefore, further analyses were focused only on the hyperthermal and hyposaline treatments.

#### Hyperthermal experiment

**Fig. 6:**
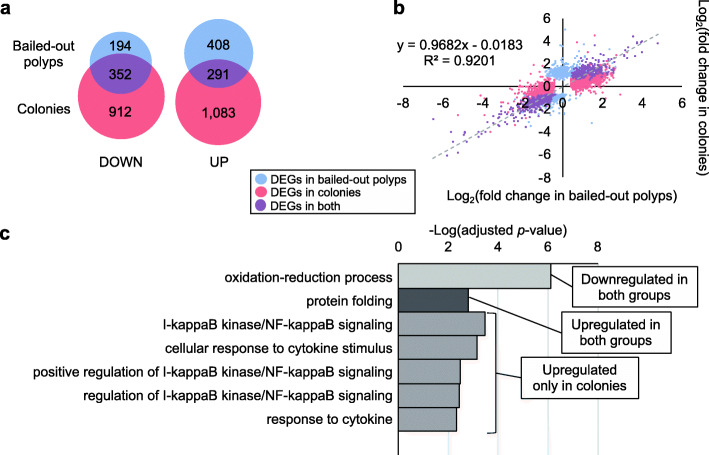
Genetic responses of bailed-out polyps and colonies during hyperthermal treatment. (**a**) Numbers of DEGs in bailed-out polyps (blue) and colonies (red). (**b**) Gene expression changes in colonies (x-axis) and bailed-out polyps (y-axis) under the treatment. DEGs specific to bailed-out polyps, to colonies, or to both, are labeled in blue, red, or purple, respectively. A regression line is fit to DEGs occurring simultaneously in both bailed-out polyps and colonies. (**c**) Selected GO terms overrepresented among DEGs specific to both bailed-out polyps and colonies (upregulation and downregulation) and among DEGs upregulated specifically in colonies

Under hyperthermal treatment, 1,245 DEGs (699 upregulated and 546 downregulated) were identified in bailed-out polyps and 2,638 (1,374 upregulated and 1,264 downregulated) occurred in colonies (Fig. 6a; A complete list of DEGs is provided in Additional file 5). Among all DEGs identified in this experiment, 20% occurred in both morphotypes (291 upregulated and 352 downregulated) and showed generally similar magnitudes of expression changes (Fig. 6b). GO analysis of these overlapping DEGs showed upregulation of *protein folding* and downregulation of *oxidation-reduction process* (Fig. 6c; A complete list of enriched GO categories is provided in Additional file 6). On the other hand, DEGs unique to colonies overrepresented biological processes such as *I-kappaB kinase/NF-kappaB signaling* and *response to cytokine* (Fig. 6c). No specific GO enrichment was identified in DEGs unique to bailed-out polyps.

#### Hyposaline experiment

**Fig. 7:**
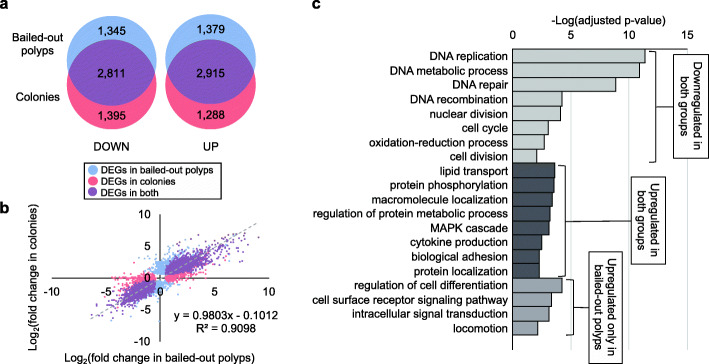
Genetic responses of bailed-out polyps and colonies during hyposaline treatment. (**a**) Numbers of DEGs in bailed-out polyps (blue) and colonies (red). (**b**) Gene expression changes in colonies (x-axis) and bailed-out polyps (y-axis) under the treatment. DEGs specific to bailed-out polyps, to colonies, or to both, are labeled in blue, red, or purple, respectively. A regression line is fit to DEGs occurring simultaneously in both bailed-out polyps and colonies. (**c**) Selected GO terms overrepresented among DEGs specific to both bailed-out polyps and colonies (upregulation and downregulation) and among DEGs upregulated specifically in bailed-out polyps

In response to hyposalinity, 8,450 DEGs (4,294 upregulated and 4,156 downregulated) were identified in bailed-out polyps and 8,409 DEGs (4,203 upregulated and 4,206 downregulated) were identified in colonies (Fig. 7a; A complete list of DEGs is provided in Additional file 7). In comparison to the hyperthermal experiment, a higher proportion of DEGs (51%) occurred in both morphotypes. Expression changes of these overlapping DEGs were generally consistent between the two groups (Fig. 7b). GO analysis showed that both bailed-out polyps and colonies responded to hyposalinity by suppressing *DNA replication* and by activating *lipid transport* and *protein phosphorylation* (Fig. 7c; A complete list of enriched GO categories is provided in Additional file 8). On the other hand, biological processes such as *regulation of cell differentiation*, *intracellular signal transduction*, *locomotion*, and *cell surface receptor signaling pathway* were overrepresented among DEGs specific to bailed-out polyps (Fig. 7c). No GO terms were significantly enriched among DEGs specific to colonies.

## Discussion

Life cycles of many stony corals involve complex dynamics of solitary and colonial lifestyles. During typical ontogeny of colonial corals, budding causes a solitary primary polyp to transition to a colonial form, which can revert to a solitary morphology through gamete production or asexually via fragmentation or polyp bail-out. An important feature of coloniality in the Scleractinia is formation of an integrated gastrovascular system, which serves functions of molecular transport, cellular component transport, and neural transmission [6–8]. Based on evidence from dye injection experiments and scanning electron microscopy, fluid circulation in coral colonies travels through the joint coelenteron between polyps and a canal system (gastrovascular canals), which ramifies through the calcified skeletons that support soft tissues [7]. Consistent with these observations, our transcriptomic analysis in *P. acuta* showed that colonies differ from bailed-out polyps by genetically activating development of tube-like structures and a nervous system, and movement of cellular components. On the other hand, compared to normal colonies, bailed-out polyps of *P. acuta* significantly activate protein synthesis. This genetic change concurs with that observed in *Oculina patagonica*, in which solitary polyps resulted from acidification-induced colony dissociation showed individual polyp biomass three times higher than that of colonial polyps in a control group [17]. These findings provide the first genetic support for the transition between colonies and bailed-out polyps and suggest a possible trade-off between polyp growth and development of a gastrovascular system. However, coral polyps in a colony may possess different morphological or physiological features, such as size, fecundity, or presence of sweeper tentacles [31–33], which may further differentiate bailed-out polyps and colonies. A solid conclusion therefore necessitates more comprehensive studies.

In addition, compared to bailed-out polyps, colonies show overexpression of several ACE-like and ECE-like genes under ambient conditions. ACE and ECE are zinc metalloproteases that show broad substrate specificities [34–36]. Homologs of vertebrate ACE and ECE have been identified in insects, cnidarians, and even in some prokaryotes, indicating early phylogenetic emergence of these enzymes [37–44]. Sequence similarity to mammalian ACE and ECE genes and the presence of a *HEXXH* motif, a conserved structural element important for metal binding by metalloproteases, suggest the existence of functional ACE and ECE homologs in *P. acuta*. By hydrolyzing angiotensin I and endothelin precursor to their corresponding active forms, respectively, ACEs and ECEs mediate vascular tone in mammals. However, blood pressure regulation is clearly not the function of these enzymes in organisms with open circulatory systems. Indeed, sequence variations in the ECE substrate recognition site (the *NAYY* motif) implies that these *P. acuta* ECE-like proteins have different substrate specificities than their mammalian homologs. Concurrent expression of *Hydra* ECE with foot and tentacle regeneration suggests a possible developmental role of ECE in the Cnidaria [40]. In insects, ACE has been proposed to participate in several non-vascular functions, such as reproduction, development, innate immunity, and prohormone processing [41]. Expression of ACE and ECE in jellyfish tentacles also points toward a role for these enzymes in envenomation [37, 45]. Adding to these studies, our findings suggest possible involvement of *P. acuta* ACEs and ECEs in the transition between polyps and colonies. However, our knowledge of ACE and ECE in cnidarians, particularly in the Scleractinia, is still negligible. Functional characterization of these enzymes is required for a more comprehensive understanding of their functions in corals.

With the ability to regulate molecular transport and signaling between polyps, coloniality has been hypothesized to facilitate coral stress responses [10, 11, 46]. Supporting this hypothesis, among DEGs overexpressed in colonies relative to bailed-out polyps under non-stressful conditions, we identified enrichment of biological processes related to interactions between corals and the environment, both internal and external. Furthermore, in both our hyperthermal and hyposaline experiments, many identified DEGs were specific to either colonies or bailed-out polyps. These results indicate that the transition between colonies and bailed-out polyps results in significant changes to coral responses to environmental stimuli. Notably, under hyperthermal stress, activation of *NF-κB signaling* occurred in colonies, but not in bailed-out polyps. Innate immunity serves as the main anthozoan defense system against pathogenic microorganisms [47–49]. Many studies have linked thermal stress-induced coral death to immunosuppression and bacterial infections [50–52]. Recently, the NF-κB gene in a stony coral, *Orbicella faveolata*, was functionally characterized, and shows high similarity to mammalian NF-κB in terms of protein structure, processing mechanisms, and DNA binding [53]. Activation of the NF-κB pathway in *O. faveolata* in response to gram-negative lipopolysaccharide treatment provides strong support for a role of the NF-κB pathway in coral innate immunity [53]. Our results thus suggest that *P. acuta* colonies may present stronger regulation of coral-microbiome interactions in hyperthermal environments, than bailed-out polyps. However, beside the transition of coloniality, bailed-out polyps differ from colonial polyps in the absence of a skeleton, which minimizes the surface area exposed to the external environment. During stress events, stony corals retract polyps into corallites, the skeletal cavities beneath individual polyps [54]. This behavioral adaptation further reduces exposure and may influence coral responses to environmental stimuli, such as elevated temperatures, high insolation, and salinity changes [16, 54–58]. Thus, a possible contribution of calcareous skeletons to the genetic differences identified in this study cannot be excluded. Future studies of other coloniality transition processes, either along the typical solitary-to-colonial ontogeny or the reverse, will be required to further test the functional association of genetic changes identified in this study and the biology of coral coloniality.

## Conclusions

**Fig. 8:**
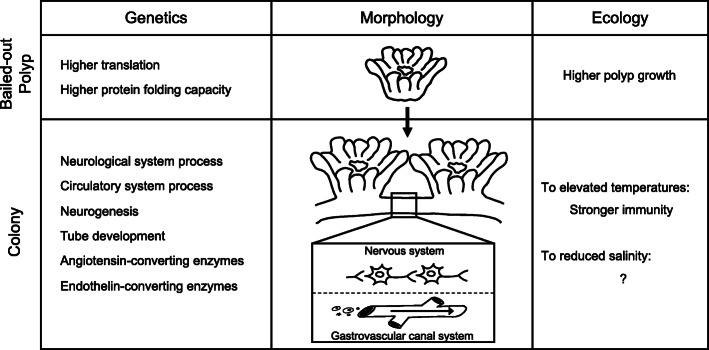
Summary of morphological, genetic, and possible ecological features of bailed-out polyps and colonies

Using bailed-out polyps of *P. acuta* as a model, this study provides the first genetic background underlying formation of the coral gastrovascular system (Fig. 8). Overexpression of ACE and ECE in colonies compared with bailed-out polyps suggests possible participation of these enzymes in colony development in *P. acuta*, warranting functional characterization of these genes in corals. The transition from bailed-out polyps to colonies, suggested by our genetic data, results in different responses to environmental changes, which presumably enhance coral fitness. This study provides additional evidence for the selective advantages of coral coloniality. Future studies with more coral representatives and other aspects of coloniality, such as budding and fragmentation, should provide further insights into the biology of coloniality in stony corals.

## Methods

### Coral sampling

In 2018 and 2019, we purchased *Pocillopora acuta* corals from the Onna Village Fisheries Cooperative in Okinawa, Japan, which collected them in shallow waters along the western coast of Okinawa. Coral colonies were visually examined for morphological integrity. To reduce possibility of duplicated samples from fragmentation, only colonies showing intact gross appearance were employed in follow-up experiments (*N* = 11). All coral colonies were acclimated in an outdoor, open-system tank for over 4 months prior to experiments. Taxonomy of each colony was confirmed by genotyping using the method and primer pair (FATP6.1 and RORF) described earlier [59].

### Polyp bail-out induction and experimental design

**Fig. 9:**
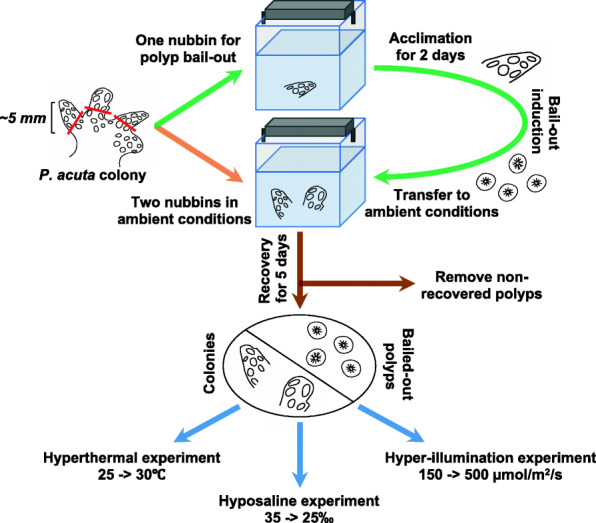
Schematic representation of the experimental design. RNA samples were collected before and after the three experiments (blue arrow) for Illumina RNA sequencing and follow-up DE analyses

For each experiment, three nubbins (~5 mm) from a colony were transferred to each of two 5-L indoor tanks, with one nubbin in a tank destined for bail-out induction and the other two in the other tank for ambient conditions. Given that polyps at different positions of a colony may vary in terms of size and developmental stage [31], coral nubbins were all removed from branch tips to reduce variation between nubbins. Cultivation was conducted in filtered natural seawater (FSW; 33–35‰) at 25 ± 0.5 °C and light was provided at 150 µmol/m^2^/s from 0600 to 1800 for a 12 h:12 h day-night cycle. Two days after transferring them, we induced polyp bail-out in one tank with a hypersaline stress and transferred bailed-out polyps to the tank of ambient conditions for recovery, following the procedure described previously [28]. Five days after bail-out, polyps showing recovered morphology were subjected to one of three treatments, along with the two healthy nubbins from the same mother colony: hyperthermal (heating from 25 to 30 °C in 10 min), hyposalinity (transfer to 25‰ FSW pre-diluted with fresh water), or hyper-illumination (change of light intensity to 500 µmol/m^2^/s). Each treatment was conducted for 6 h during the daytime (1000–1600) and was repeated with three different colonies. In each experiment, bailed-out polyps (5–10 polyps/sample) and normal colonies (1 nubbin/sample) were collected both before and after treatment and preserved immediately in 1 mL TRIZOL reagent. A schematic of the experimental design is presented in Fig. 9.

### RNA extraction and sequencing

RNA extraction was performed following a TRIZOL RNA extraction protocol [60]. Extracted RNA samples were checked for RNA quality (RNA integrity number ≥ 7) and were used for Illumina RNAseq library construction (with polyA-purification and strand specification), performed by the DNA Sequencing Section at the Okinawa Institute of Science and Technology (OIST). Paired-end RNA sequencing (150 × 150 bp) was then performed on an Illumina Novaseq 6000 Sequencing System.

### De novo transcriptome assembly

RNAseq libraries sequenced in this study and those sequenced in our previous reports (Additional file 9) [23, 28] were pooled for *de novo* transcriptome assembly using Trinity v2.8.4 [61]. The constructed transcriptome assembly was first filtered to remove transcripts < 200 bp and transcripts sharing > 95% similarity were clustered using CD-HIT-EST [62]. Transcript quantification was conducted using RSEM v1.3.2 [63] and those with < 5 transcripts per million (TPM) or showing possible contamination (identified by the Contamination Screen in the NCBI database) were removed, generating a coral holobiont meta-transcriptome of 188,132 transcripts (N50 length: 2,149 bases).

### Coral gene identification

From the ReefGenomics database and the Marine Genomics Unit at OIST, we first downloaded three genomes and four transcriptome assemblies of pocilloporid corals and six genome assemblies of symbiotic zooxanthellae (Additional file 10) [64, 65]. Predicted proteins from these genomes and transcriptome assemblies were pooled to constructed a local database, representing five pocilloporid corals and four genera of zooxanthellae in the Symbiodiniaceae, based on recently revised systematics [66]. The coral holobiont meta-transcriptome was then BLAST searched against this local database. Transcripts best matched to pocilloporid coral proteins (E-value < 10^− 5^) were selected to create a transcriptome assembly of the coral host, *P. acuta* (65,935 transcripts; N50: 3,433 bases; GenBank accession: GJAW00000000), which showed 90.6% completeness in a BUSCO analysis using the metazoa_odb9 dataset [67].

### Differential gene expression analysis

For a comparative analysis of bailed-out polyps and normal colonies, transcript quantification was conducted again for RNAseq libraries constructed in this study (Additional file 9) against the *P. acuta* transcriptome assembly, using RSEM. DE analyses were conducted for pairwise comparisons of RNAseq libraries before and after treatments in the three experiments, with three biological replicates in all conditions in each experiment. For comparisons between bailed-out polyps and colonies under ambient conditions, before-treatment RNAseq libraries in the three experiments were pooled for a DE analysis, with nine datasets in each condition, representing six biological replicates (three colonies were employed in multiple experiments). All DE analyses were conducted using edgeR in the Trinity pipeline [68], with criteria for a DEG set as the false discovery rate (FDR) < 0.01 and > 5 counts per million (CPM) in at least one-third of RNAseq libraries in the pairwise comparison. ORFs in transcripts in the *P. acuta* transcriptome assembly were predicted using TransDecoder and functional annotation was conducted based on the SwissProt eukaryotic database (E-value < 10^− 5^) using Trinotate [69]. DAVID bioinformatics resources v6.8 were then applied to examine GO enrichment among DEG subsets of interest [70, 71]. Statistical significance of GO enrichment was determined using a Bonferroni-adjusted *p*-value < 0.01.

### Quantitative polymerase chain reaction

Gene expression in bailed-out polyps and normal colonies was confirmed by qPCR. The polyp bail-out process was repeated on five additional *P. acuta* colonies and bailed-out polyps and nubbins under ambient conditions were collected five days after bail-out. RNA extraction was followed by cDNA synthesis using SuperScript IV VILO Master Mix (Invitrogen, USA). Three ACE-like and three ECE-like genes were included in a qPCR assay, with a ß tubulin gene and an actin gene (both showed no significant differential expression between bailed-out polyps and colonies in the transcriptomic data) serving as internal control genes. qPCR was conducted on a StepOnePlus real-time PCR system following the procedure described previously [23]. Relative gene expression was calculated as the difference of cycle thresholds (ΔCT) between the target gene and an average of the two internal control genes: ΔCT = CT_average of control genes_ – CT_target gene_. Statistical significance of gene expression differences between colonies and bailed-out polyps was determined by a *p*-value < 0.05 using a paired sample *t*-test. All primers used in this study are listed in Table 3.

**Table 3:** Primers employed in the qPCR assay. Reference transcripts in the *P. acuta* transcriptome assembly are listed. *Primers for the ß tubulin are adopted from a previous study

Functional annotation	Primer sequence (5’ – 3’)	Reference transcript
Angiotensin-converting enzyme	f: gataaacagcagcgggaag r: agattcggtgacaaagacaag	TRINITY_DN5954_c0_g3_i1
	f: actttctctgaaccccgac r: atccctccaccattccttcc	TRINITY_DN10767_c0_g1_i10
	f: caagtggatgatggaacagag r: agtgttgaacagtgtgggaag	TRINITY_DN3110_c4_g2_i4
Endothelin-converting enzyme	f: cggaacatcaagcacagag r: aaaggacggtaatcaacacag	TRINITY_DN4184_c0_g1_i8
	f: ctactcacccaggcaaaatc r: ccaatcaccataccaattccac	TRINITY_DN14970_c0_g1_i2
	f: tgactccccccactgtaaac r: ccaacaaccattccgattcc	TRINITY_DN36866_c0_g1_i3
Actin	f: tgtctcgatcaataaaccttcc r: cccataccaaccatcactcc	TRINITY_DN21309_c1_g1_i1
ß tubulin	f: gcagttcacggctatgttc* r: ttttcaccctcctcttcctc*	Chuang and Mitarai (2020)

### Sequence alignment and structure analysis

Protein sequences deduced from predicted ORFs in three *P. acuta* ACE-like and three ECE-like genes were analyzed with known ACE (*Homo sapiens*: AAA60611; *Mus musculus*: AAA37149; *Drosophila melanogaster*: AAB02171) and ECE (*Homo sapiens*: BAA07800; *Mus musculus*: NP_001356106; *Danio rerio*: NP_001071260; *Hydra vulgaris*: AAD46624) proteins of other organisms, downloaded from the NCBI protein database. Sequence alignment was conducted using MEGA X [72] and functional domains in each protein were predicted using InterPro [73]. Sequence identities between aligned regions of *P. acuta* ACE-like and ECE-like proteins with ACE and ECE proteins from other organisms, respectively, were calculated using the Ident and Sim program in the Sequence Manipulation Suite [74].

## Supplementary Information

**Additional file 1.** DEGs between bailed-out polyps and colonies. Result of a DE analysis between bailed-out polyps (N = 9) and normal colonies (N = 9) under ambient conditions. Transcripts are presented as transcriptID^annotation. P-values and FDR for expression differences between the two groups are presented for each transcript. Expression levels are presented as TPM after trimmed mean of M values (TMM) cross-sample normalization. RNAseq libraries are presented as treatment (Hyperthermal/Hyper-illumination/Hyposalinity)_coral morphotype (Colony/Polyp)_biological replicate (rep1/rep2/rep3).

**Additional file 2.** GO enrichment among DEGs between bailed-out polyps and colonies. Enriched biological processes among DEGs between bailed-out polyps and normal colonies under ambient conditions. All biological processes with Bonferroni-adjusted *p*-values < 0.05 are listed with corresponding fold enrichment values.

**Additional file 3.** ORFs of three ACE-like genes in P. acuta. Corresponding transcript IDs are provided in parentheses.

**Additional file 4.** ORFs of three ECE-like genes in P. acuta. Corresponding transcript IDs are provided in parentheses.

**Additional file 5.** DEGs in bailed-out polyps and colonies in the hyperthermal experiment. Results of DE analyses in bailed-out polyps (N = 3) and colonies (N = 3) in response to hyperthermal treatment. Transcripts are presented as transcriptID^annotation. P-values and FDR for expression difference before and after the treatment are presented for each transcript. Expression levels are presented as TPM after TMM cross-sample normalization. RNAseq libraries are presented as coral morphotype (Colony/Polyp)_sampling time (pre/after)_biological replicate (rep1/rep2/rep3).

**Additional file 6.** GO enrichment among DEGs in the hyperthermal experiment. Enriched biological processes among DEGs identified simultaneously in bailed-out polyps and colonies, specific to bailed-out polyps, or specific to colonies are listed separately. All biological processes with Bonferroni-adjusted p-values < 0.05 are listed with corresponding fold enrichment values.

**Additional file 7.** DEGs in bailed-out polyps and colonies in the hyposaline experiment. Results of DE analyses in bailed-out polyps (N = 3) and colonies (N = 3) in response to hyposaline treatment. Transcripts are presented as transcriptID^annotation. P-value and FDR for expression difference before and after the treatment are presented for each transcript. Expression levels are presented as TPM after TMM cross-sample normalization. RNAseq libraries are presented as coral morphotype (Colony/Polyp)_sampling time (pre/after)_biological replicate (rep1/rep2/rep3).

**Additional file 8.** GO enrichment among DEGs in the hyposaline experiment. Enriched biological processes among DEGs identified simultaneously in bailed-out polyps and colonies, specific to bailed-out polyps, or specific to colonies are listed separately. All biological processes with Bonferroni-adjusted p-values < 0.05 are listed with corresponding fold enrichment values.

**Additional file 9.** RNAseq libraries used to construct the P. acuta transcriptome assembly. Morphologies and cultivation conditions of the samples are presented. Libraries employed in DE analyses are highlighted in bold. *Six libraries were constructed as part of the present study, but were first reported in a previous study.

**Additional file 10.** Genomic and transcriptomic databases applied to identify coral transcripts. Numbers of proteins in the databases are indicated. Species names of zooxanthellae are based on a recently revised taxonomy.

## Data Availability

Transcriptome assembly and RNAseq datasets constructed in this study can be found in the NCBI GenBank database under the following accession numbers and link: GJAW00000000, SRR12639766-SRR12639772, and SRR13743385-SRR13743414 (https://www.ncbi.nlm.nih.gov/nuccore/GJAW00000000).
