# Supplementary material for: Genetic changes involving the coral gastrovascular system support the transition between colonies and bailed-out polyps: evidence from a Pocillopora acuta transcriptome
Source: BMC Genomics. 2021 Sep 26;22:694. doi: 10.1186/s12864-021-08026-x (PMC8466926; doi:10.1186/s12864-021-08026-x)
Supplement: Supplementary file 9 — Additional file 9. RNAseq libraries used to construct the P. acuta transcriptome assembly. Morphologies and cultivation conditions of the samples are presented. Libraries employed in DE analyses are highlighted in bold. *Six libraries were constructed as part of the present study, but were first reported in a previous study. [file 12864_2021_8026_MOESM9_ESM.docx]

Additional file 9. RNAseq libraries used to construct the *P. acuta* transcriptome assembly.

| **Accession #** | **Size** | **Condition** | **Source** |
| --- | --- | --- | --- |
| SRR10696826  SRR10708185  SRR10708225 | 11.7G  7.2G  13.8G | Colony  25˚C; 300 µmol/m^2^/s; 35‰  (Polyp bail-out; treatment group) | Chuang and Mitarai (2020) |
| SRR10696825  SRR10708184  SRR10708231 | 11.8G  14.5G  14.5G | Colony  25˚C; 300 µmol/m^2^/s; 43‰  (Polyp bail-out; treatment group) | Chuang and Mitarai (2020) |
| SRR10696824  SRR10708183  SRR10708230 | 10.8G  14.3G  15.2G | Colony  25˚C; 300 µmol/m^2^/s; 46‰  (Polyp bail-out; treatment group) | Chuang and Mitarai (2020) |
| SRR10696823  SRR10708182  SRR10708229 | 11.9G  14.1G  6.8G | Bailed-out polyp  25˚C; 300 µmol/m^2^/s; 46‰  (Polyp bail-out; treatment group) | Chuang and Mitarai (2020) |
| SRR10696829  SRR10708188  SRR10708228 | 11.7G  12.8G  12.3G | Colony  25˚C; 300 µmol/m^2^/s; 35‰  (Polyp bail-out; control group) | Chuang and Mitarai (2020) |
| SRR10696828  SRR10708187  SRR10708227 | 11.5G  14.8G  13.8G | Colony  25˚C; 300 µmol/m^2^/s; 35.4‰  (Polyp bail-out; control group) | Chuang and Mitarai (2020) |
| SRR10696827  SRR10708186  SRR10708226 | 14.2G  14.6G  13.7G | Colony  25˚C; 300 µmol/m^2^/s; 35.8‰  (Polyp bail-out; control group) | Chuang and Mitarai (2020) |
| SRR12639762  SRR12639763  SRR12639764  SRR12639765  SRR12639770 | 25.3G  26G  24.6G  25.8G  16.1G | Bailed-out polyp  25˚C; 150 µmol/m^2^/s; 35‰  (Five days post-bail-out; morphologically degenerated) | Chuang et al. (2021) |
| **SRR12639766***  **SRR12639768***  **SRR12639771*** | 9.1G  8.7G  8.9G | Bailed-out polyp  25˚C; 150 µmol/m^2^/s; 35‰  (pre-hyperthermal treatment) | Chuang et al. (2021) |
| **SRR13743413**  **SRR13743391**  **SRR13743389** | 7.9G  8.2G  8.1G | Bailed-out polyp  30˚C; 150 µmol/m^2^/s; 35‰  (post-hyperthermal treatment) | This study |
| **SRR12639767***  **SRR12639769***  **SRR12639772*** | 7.8G  7.5G  8.8G | Colony  25˚C; 150 µmol/m^2^/s; 35‰  (pre-hyperthermal treatment) | Chuang et al. (2021) |
| **SRR13743414**  **SRR13743402**  **SRR13743390** | 8.6G  8.2G  8.7G | Colony  30˚C; 150 µmol/m^2^/s; 35‰  (post-hyperthermal treatment) | This study |
| **SRR13743410**  **SRR13743406**  **SRR13743386** | 12.8G  11.9G  11.9G | Bailed-out polyp  25˚C; 150 µmol/m^2^/s; 35‰  (pre-hyper-illuminated treatment) | This study |
| **SRR13743409**  **SRR13743405**  **SRR13743385** | 14.4G  11.6G  12.6G | Bailed-out polyp  25˚C; 500 µmol/m^2^/s; 35‰  (post-hyper-illuminated treatment) | This study |
| **SRR13743412**  **SRR13743408**  **SRR13743388** | 13.6G  11.7G  12.0G | Colony  25˚C; 150 µmol/m^2^/s; 35‰  (pre-hyper-illuminated treatment) | This study |
| **SRR13743411**  **SRR13743407**  **SRR13743387** | 12.5G  11.0G  13.8G | Colony  25˚C; 500 µmol/m^2^/s; 35‰  (post-hyper-illuminated treatment) | This study |
| **SRR13743401**  **SRR13743397**  **SRR13743393** | 12.2G  11.9G  12.3G | Bailed-out polyp  25˚C; 150 µmol/m^2^/s; 35‰  (pre-hyposaline treatment) | This study |
| **SRR13743400**  **SRR13743396**  **SRR13743392** | 11.0G  11.8G  16.0G | Bailed-out polyp  25˚C; 150 µmol/m^2^/s; 25‰  (post-hyposaline treatment) | This study |
| **SRR13743404**  **SRR13743399**  **SRR13743395** | 13.0G  11.9G  11.7G | Colony  25˚C; 150 µmol/m^2^/s; 35‰  (pre-hyposaline treatment) | This study |
| **SRR13743403**  **SRR13743398**  **SRR13743394** | 11.9G  11.9G  13.0G | Colony  25˚C; 150 µmol/m^2^/s; 25‰  (post-hyposaline treatment) | This study |

Morphologies and cultivation conditions of the samples are presented. Libraries employed in DE analyses are highlighted in bold. *Six libraries were constructed as part of the present study, but were first reported in a previous study.
