# Supplementary material for: Genetic changes involving the coral gastrovascular system support the transition between colonies and bailed-out polyps: evidence from a Pocillopora acuta transcriptome
Source: BMC Genomics. 2021 Sep 26;22:694. doi: 10.1186/s12864-021-08026-x (PMC8466926; doi:10.1186/s12864-021-08026-x)
Supplement: Supplementary file 10 — Additional file 10. Genomic and transcriptomic databases applied to identify coral transcripts. Numbers of proteins in the databases are indicated. Species names of zooxanthellae are based on a recently revised taxonomy. [file 12864_2021_8026_MOESM10_ESM.docx]

Additional file 10. Genomic and transcriptomic databases applied to identify coral transcripts.

| **Pocilloporid corals** | | | |
| --- | --- | --- | --- |
| **Species** | **Type** | **# of proteins** | **source** |
| *Pocillopora damicornis* | Genome | 26077 | ReefGenomics |
| *Pocillopora damicornis* | Transcriptome | 20509 | ReefGenomics |
| *Pocillopora verrucosa* | Genome | 27439 | ReefGenomics |
| *Stylophora pistillata* | Genome | 25769 | ReefGenomics |
| *Stylophora pistillata* | Transcriptome | 21810 | ReefGenomics |
| *Seriatopora hystrix* | Transcriptome | 27680 | ReefGenomics |
| *Seriatopora sp.* | Transcriptome | 35409 | ReefGenomics |
| **Symbiodiniaceae** | | | |
| **Species** | **Type** | **# of proteins** | **source** |
| *Symbiodinium microadriaticum* | Genome | 49109 | ReefGenomics |
| *Cladocopium goreaui* | Genome | 35913 | ReefGenomics |
| *Fugacium kawagutii* | Genome | 26609 | ReefGenomics |
| *Symbiodinium sp.* (clade A3) | Genome | 90078 | Shoguchi et al. (2018) |
| *Breviolum minutum* | Genome | 47014 | Shoguchi et al. (2013) |
| *Cladocopium sp.* (clade C) | Genome | 79161 | Shoguchi et al. (2018) |

Numbers of proteins in the databases are indicated. Species names of zooxanthellae are based on a recently revised taxonomy.
